# An Investigation of the Mechanical Properties of Flax/Basalt Epoxy Hybrid Composites from a Sustainability Perspective

**DOI:** 10.3390/polym16192839

**Published:** 2024-10-08

**Authors:** Martina Panico, Ersilia Cozzolino, Ilaria Papa, Iman Taha, Valentina Lopresto

**Affiliations:** 1 Department of Chemical, Materials and Production Engineering, University of Naples Federico II, P.le Tecchio 80, 80125 Naples, Italy; martina.panico@unina.it (M.P.); ilaria.papa@unina.it (I.P.); lopresto@unina.it (V.L.); 2 Sustainable Materials in Polymer Engineering, Aalen University, Beethovenstraße 1, 73430 Aalen, Germany; iman.taha@hs-aalen.de

**Keywords:** biocomposites, basalt, flax, epoxy resin, hybrid composite materials, sustainability, lightweight materials

## Abstract

Currently, sustainability plays a central role in the response to global challenges, strongly influencing decisions in various sectors. From this perspective, global efforts to explore inventive and eco-friendly solutions to address the demands of industrialization and large-scale production are being made. Bio-based composites needed for lightweight applications benefit from the integration of natural fibers, due to their lower specific weight compared to synthetic fibers, contributing to the overall reduction in the weight of such structures without compromising the mechanical performance. Nevertheless, challenges arise when using natural fibers in composite laminates and hybridization seems to be a solution. However, there is still a lack of knowledge in the literature regarding the strategies and possibilities for reducing laminate thickness, without sacrificing the mechanical performance. This work aims to fill this knowledge gap by investigating the possibility of reducing the laminate thickness in hybrid flax/basalt composites made of plies, organized in the same stacking sequence, through only varying their number. Tensile, Charpy, flexural, and drop-weight tests were carried out for the mechanical characterization of the composites. The results obtained confirm the feasibility of achieving thinner hybrid composites, thus contributing to sustainability, while still having acceptable mechanical properties for structural applications.

## 1. Introduction

### 1.1. Background

Fiber-reinforced plastics (FRPs) are composite materials made of a polymer matrix reinforced with fibers. They are typically used in a wide range of sectors, such as aerospace, naval, automotive, and biomedical industries. Currently, researchers are exploring innovative configurations to take advantage of the opportunity offered by combining a different stacking sequence of different materials into composite laminates [1,2]. In this regard, previous studies have investigated strategies to obtain composite materials with superior mechanical properties that meet industrial quality standards, while reducing costs and the overall environmental impact [3].

Structural engineering often utilizes composite materials and, in this regard, FRPs made with continuous fibers show excellent potential for reducing both the weight and lifetime maintenance costs associated with such materials, owing to their corrosion and fatigue resistance [4]. In FRPs used for structural applications, the polymer is typically an epoxy, vinyl ester, or polyester thermosetting plastic, whereas glass and carbon are among the most widely employed fibers used for reinforcement.

Nevertheless, in the current situation, climate change has shown that it is of paramount importance to make manufacturing processes and product life cycles more sustainable. For these reasons, in the last decade, natural fibers have gained increasing interest in the industry as a form of reinforcement in polymer composites, resulting in the creation of materials known as natural fiber composites [5]. Natural fiber can be derived from different sources, such as plants, animals, or minerals. Their low density, low cost, high level of sustainability, and superior mechanical properties are some of the main factors driving the interest in natural fiber composites. Moreover, natural fiber composites are often biodegradable and have a lower carbon footprint than their synthetic equivalents. Natural fiber composites can enhance the mechanical properties, acoustic performance, and impact energy absorption of some composites [6]. However, several drawbacks prevent natural fiber composites from being widely used. Natural fibers absorb moisture, leading to dimensional instability and deterioration of the mechanical characteristics of the composite material. Additionally, natural fiber composites often lack consistency because of the heterogeneity of natural fibers [7]. As a result, regarding the current state-of-the-art in this field, the use of carbon or glass fibers seems to be unavoidable, especially for marine, aerospace, and civil engineering industries, where structures typically operate in challenging environments and are required to offer properties such as damage tolerance and resistance to high-impact loads [8].

### 1.2. Literature Review

Considering the impact properties, natural fiber composites are currently under investigation because they still cannot compete with traditional synthetic fibers. In this context, hybridization seems to be a valid solution to overcome this issue. It is typically used to combine the advantages of two or more kinds of fibers or matrices, resulting in the performance of hybrid composites being the weighted sum of the specific constituents. Based on this premise, the hybrid design may help mitigate the weakest points of both natural and synthetic fibers, thus increasing sustainability by reducing the amount of synthetic fiber used, while increasing impact energy absorption [9]. Hybrid composites are more promising than other fiber-supported composites and have a wider range of potential applications. This is because the benefits of the different fibers boost the qualities of each other. Hence, the associated costs and performance of the materials can be optimized through the strategic design of the hybrid composite [10]. Examples of hybrid composites include the kenaf–aramid hybrid made with Kevlar [11], woven jute/glass fabric, sisal fiber-reinforced polyester composites with the addition of carbon [12], as well as silicon carbide/jute epoxy composites [13]. Hani et al. [14] investigated woven coir–Kevlar hybrid composites and found that coconut coir can replace some synthetic fibers, thereby improving the resistance of the material to high-speed impacts and penetration.

It is generally known that the fiber architecture, such as the length, orientation, content, distribution in the matrix, and shape, influence the strength of polymer composites. For instance, longer fibers typically result in higher strength in the fiber direction, whereas shorter fibers guarantee a more uniform distribution within the matrix but contribute less to the composite strength [15]. Akhyar et al. [16] investigated the effect of the coconut fiber size on the flexural strength of natural fiber composites. It was shown that the flexural strength and flexural modulus were not significantly affected by the fiber size.

The properties of a hybrid composite composed of two fibers are influenced not only by the individual fiber characteristics, but also by the layering pattern of the two fibers, their intermingling capacity, the fiber-to-matrix interface, and also the failure strain of single fibers. Thus, the design of the hybrid composite can be crucial, because the presence of more fibers can also introduce weaknesses into the material. For this reason, the constituents of the hybrid composite must be selected considering the purpose of the hybridization, the material requirements, and the construction being developed [3]. Sisal and oil palm fibers are an excellent combination in terms of hybrid composites, due to the high tensile strength of sisal fiber and the high toughness of oil palm fiber. Therefore, composites that include both sisal and oil palm fibers typically exhibit desirable properties. Hybrid composites can be used for both structural and non-structural applications and can be strategic drivers for sustainability, since they can result in up to a 50% weight decrease [17], thereby significantly minimizing CO_2_ emissions.

Sustainability needs to be considered during the consideration of the life cycle aspects of materials, as well as in terms of the manufacturability and functionality of the parts. Finding a good compromise between the required properties of the materials and minimizing the overall environmental impact can be a challenging task. As mentioned above, natural fiber composites could represent a valid solution to pursue these objectives; however, the literature still lacks substantial research needed to standardize and consider natural fiber composites as valuable substitutes for FRPs made of synthetic fibers.

Many studies in the literature have investigated the effect of the stacking sequence and hybridization on the mechanical properties of composites. Kureemun et al. [18] studied hybrid configurations with different flax/carbon ply stacking sequences, with a low carbon fiber volume fraction. They found that adding 8% carbon fiber by volume, according to the amount of woven flax fabric, significantly helps to increase the tensile strength and stiffness of the material, by up to 50%. In their work, Jusoh et al. [19] investigated the effect of sandwich-like and intercalation sequences in epoxy composites reinforced with glass/basalt, glass/jute, and glass/flax. They found a negligible effect of the stacking on the tensile properties, whereas the flexural strength and flexural modulus were significantly affected, with extensive delamination phenomena observed at the interfaces.

Among all the existing hybrid combinations that aim to act as reinforcements and those that have been investigated, basalt and flax seem to be the most promising regarding the enhancement of the resistance to low-velocity impacts. Basalt is now considered a valid alternative to conventional glass fibers. Basalt fiber is made of mineral materials; it is non-toxic, easy to process, environmentally friendly, and recyclable. The tensile strength and flexural modulus of basalt are 2600–4840 MPa and 80–115 GPa, respectively, whereas the tensile strength and flexural modulus of glass fiber are 1500 MPa and 71 GPa, respectively [20]. Thus, basalt fibers are twice as strong as glass fiber, making it more resistant to breakage and an ideal material for applications requiring high strength and stiffness. Moreover, basalt fibers do not corrode in fresh or salt water, they are naturally resistant to ultraviolet and high-energy electromagnetic radiation, maintain their properties in cold temperatures, and provide better acid resistance. In addition, it has been demonstrated that basalt fibers also represent a sustainable and cost-effective alternative to glass fibers [21].

Flax fiber is a natural, cellulosic, multi-cellular bast fiber, obtained from the inner bark of the stem of a plant grown in temperate and subtropical regions, offering several benefits. In fact, flax fiber is highly versatile because of its good mechanical properties, low density, high toughness, and strength. Thus, flax fibers are widely used in automotive industries as a form of reinforcement, especially for producing interior parts like door panels, as well as structural components, such as floors [22].

For all these reasons, some researchers [23,24] have focused their attention on the impact behavior of basalt/flax hybrid laminates within vinyl ester resin. They found an improvement in the impact performance of composites with a flax core between the basalt fiber skins. Also, Papa et al. [25] investigated the impact behavior of a reinforced epoxy composite, with flax and basalt twill layers alternately stacked, and confirmed the positive role of fiber hybridization in terms of damage occurrence. Ali et al. [26] investigated the effect of the laminate thickness on damage propagation and crack growth in plain-woven carbon fiber composites under Iosipescu shear loading. Acoustic emission, infrared thermographs, and ex situ micro-CT analyses revealed the increase in the sample thickness and its effect on the increased crack growth near the top and bottom V-notched section, due to the tension–compression configuration of the applied load. In their study, Fragassa et al. investigated flax/basalt hybrids, giving specific attention to the factual evaluation of the influence of basalt as a hybrid reinforcement in combination with flax fiber, focusing especially on the modes of damage observed as a consequence of impact energy absorption. Their results challenge the idea that basalt/flax fiber hybrid laminates only offer a good level of performance in the presence of basalt fibers at the outer layers and suggests possible adoption in the future of more complex stacking sequences, involving the intercalation of flax and basalt layers [27].

Another study on basalt/flax hybrids, where basalt fiber-reinforced layers were placed externally, demonstrated a significant improvement in the resistance under salt and fog conditions, which only partially affected the Charpy impact properties, where the basalt fiber external layers showed limited resilience under prolonged aging [28].

### 1.3. Objective

Currently, the literature lacks studies investigating the possibility of reducing the laminate thickness in hybrid flax/basalt composites by varying the number of plies, while maintaining the stacking sequence. This work aims to fill this gap in the knowledge. In particular, the purpose of this study is to investigate the possibility of reducing the thickness of hybrid composites reinforced with flax and basalt, and from a sustainability perspective, the possibility of reducing the weight of the materials, thus reducing the CO_2_ emissions during the manufacturing process, as well as the use and disposal phases in terms of laminates fabricated by Vacuum-Assisted Resin Infusion (VARI), all without sacrificing the mechanical properties. To pursue this aim, the stacking sequence was fixed, while the thickness and, accordingly, the number of plies, was varied. Tensile, flexural, Charpy, and drop-weight tests were carried out to mechanically characterize the hybrid laminates. Finally, the study thoroughly explains and discusses the potential for reducing the number of plies, while fixing all the other fabrication conditions for hybrid flax/basalt laminates. The conclusions and the perspective pursued represent the novelty of this work.

## 2. Materials and Methods

### 2.1. Materials and Laminate Fabrication

In this study, a hybrid intraply flax–basalt twill 2/2 woven fabric, with an areal density of 250 g/m^2^ and an orientation of 0°/90°, produced by Isomatex (Gembloux, Belgium), was used. The fabric uses 50% FILAVA^TM^, a direct roving, composed of enhanced volcanic rock filaments, manufactured using the melt spinning process and further enriched with various minerals, combined with 50% flax fibers. The matrix resin used was GreenPoxy 56, developed by Sicomin Composites (Pluguffan, France), with up to 56% of its molecular structure derived from renewable plant sources. It exhibits excellent wetting of the reinforcements and is ideal for vacuum bagging techniques involving composite laminates, as per the manufacturer’s datasheet [29]. The hardener utilized for the resin system was SD 477X, also supplied by Sicomin Composites. The resin and hardener were mixed using a drill mixer, with a weight ratio of 100:30, where the ratio denotes the mass of resin to the mass of hardener. Three laminates of 670 mm × 300 mm were manufactured, differing in terms of the number of plies used, namely 6, 9, and 12 (labelled as IP_6_, IP_9_, and IP_12_, respectively). The laminates were constructed by stacking the individual plies, thereby preserving the orientation of each ply. Consequently, the orientation of the fibers in each laminate remained consistent with the original 0/90° fabric orientation.

The design characteristics of the laminates are summarized in Table 1. The fiber mass fraction (FMF) value observed in the laminates under study, namely 44.3% for [IP_6_], 46.3% for [IP_9_], and 47.3% for [IP_12_], are consistent with the data reported in the literature for laminates produced using Vacuum-Assisted Resin Infusion [30]. These values align with the expected range for natural fiber-reinforced composites, where the FMF tends to be lower compared to synthetic fiber-reinforced laminates [31]. This is primarily due to the intrinsic discontinuity of natural fibers and their lofting nature, which reduces the packing density and limits the fiber volume fraction.

The technology employed to produce the laminates was Vacuum-Assisted Resin Infusion (VARI). The process ensures the production of high-quality, low-porosity composites, when through-thickness pathways and prepregs are employed [32,33]. 

The intraply fabric was initially cut into 6, 9, and 12 rectangular pieces for the IP_6_, IP_9_, and IP_12_ configurations, respectively. To enhance the fiber impregnation and reduce the moisture content, the rectangular pieces were dried in a Memmert ULE 700 oven (Schwabach, Germany), at 90 °C, for 30 min. For each configuration, a stack of dry reinforcement plies was laminated over a rigid mold, after being thoroughly cleaned and treated with release agent. Vacuum bagging sealant tape was then applied to the plate, creating a slightly oversized rectangular frame. A peel ply layer and flow aid were added to ensure uniform resin distribution and for the removal of excess resin and air bubbles. On the peel ply, a flow media breather was applied to control the distribution of the resin flow. The entire setup was enclosed in a vacuum bag, which was sealed to ensure airtightness. Vacuum pressure was applied, creating a vacuum within the bag. The resin was then introduced into the mold through an inlet tube, driven by the vacuum pressure to impregnate the laminate. Post-infusion, the laminate was cured for 24 h.

Finally, the resulting laminates were cut using a waterjet cutting machine to produce specimens with specific dimensions required for the subsequent static and dynamic characterization.

### 2.2. Experimental Tests

#### 2.2.1. Tensile Test

Tensile tests were conducted on five specimens using a ZwickRoell (Ulm, Germany) Z100 universal testing machine, fitted with a 100 kN load cell. The tests followed the DIN EN ISO 527-4 type 4 standard [34] and were performed by applying a uniaxial tensile load at a constant crosshead speed of 2 mm/min. This standard also specifies the geometry of the specimen, including the gradual increase in width towards the ends to ensure uniform stress distribution and minimize stress concentrations. Therefore, the specimens featured a rectangular geometry measuring 300 mm × 25 mm, with a central section that gradually expanded to 28 mm at the ends.

The tensile strength σ_m_, elongation at break ε_max_, and tensile modulus *E*, were evaluated for all the specimens.

#### 2.2.2. Flexural Test

Flexural tests were conducted using a QUASAR 50 Galdabini (Cardano al Campo, Varese) testing machine, equipped with a 50 kN load cell, in accordance with the ASTM D790 standard [35]. The test specimens, each measuring 100 mm × 15 mm, were subjected to bending, with a fixed span length of 80 mm. The crosshead speed was set to 4 mm/min, according to the standard. A total of five specimens was tested to assess the flexural strength σ_f_, the flexural modulus of elasticity *E*_f_, and the maximum strain to failure ε_f_,_max_.

#### 2.2.3. Charpy Test

Charpy impact tests were conducted on five specimens for each laminate configuration, using a ZwickRoell impact testing machine, following the DIN EN ISO 179-1 [36] standard. The specimens, with rectangular dimensions of 10.2 mm × 2.60 mm, included the IP_6_, IP_9_, and IP_12_ types. For the IP_6_ and IP_9_ specimens, a hammer with an impact energy of 7.5 J was used, considering the energy of air resistance equal to 0.01 J. Whereas, the IP_12_ specimens were impacted with a 15 J hammer, accounting for an air resistance energy of 0.03 J. The specimens were impacted according to a flatwise position.

The primary output of this testing procedure was the assessment of the impact strength [kJ/m^2^] to determine the toughness and resistance to impact-related failures of the specimens. The impact strength was calculated as the ratio between the energy absorbed [J], which was measured by the impact testing machine, and the cross-sectional area of the specimen.

#### 2.2.4. Drop-Weight Test

Both penetration and indentation tests were carried out at different levels of impact energy I_e_, by a Ceast Instron Fractovis falling weight machine (Darmstadt, Germany), according to the ASTM D7137 standard [37]. The machine was equipped with a digital acquisition system that allows an impact velocity in the range of 2–20 m/s, with the impactor mass ranging between 3.6 and 10 kg. In particular, the penetration tests were carried out at U = 100 J to evaluate the overall effect of fiber reinforcement, while the indentation tests were carried out to provide information related to the start and evolution of the damage. Accordingly, impact energy values that correspond with characteristic points, such as the load drop and changes in the pendency of the penetration curve’s linear part, were evaluated. Table 2 provides the details of the penetration and indentation impact energies used for each laminate configuration.

For both the penetration and indentation tests, square specimens of 100 mm × 100 mm were used, one for each level, for each laminate configuration. Each square specimen was clamped and centrally loaded by an instrumented cylindrical impactor, with a hemispherical nose that was 19.8 mm in diameter and had a total mass of 3.64 kg. The impactor was equipped with sensors to measure the force values, while a probe on the machine recorded the velocity. This data enabled the generation of the load–displacement curve and the calculation of the absorbed energy post-impact, facilitating the assessment of the effectiveness of increasing the number of plies in enhancing the toughness of the material.

The indentation depth *I* was measured using a LEXT OLS5000 confocal microscope Olympus (Segrate, Italy), to gain further information related to the damage achieved at all the investigated levels of impact energy. The microscope is equipped with different magnifications (5–150 X), an x-y table, and dedicated software (OLS5000 ver. 1.3.5 software). For each acquisition, three measurements of the damage depth were taken and the average of these three values was reported.

To analytically study the relationship between the test outputs and the laminate thickness variable, an ANOVA and Tukey’s method were employed, using the MINITAB 21 software [38]. These statistical analyses were employed to determine whether significant differences existed among the groups and to identify specific group differences with a 95% confidence interval. The results will be discussed based on the F-values and *p*-values derived from the ANOVA, along with the groupings indicated by the Tukey test, providing a comprehensive understanding of the variability and significance of the test outputs in relation to the laminate thickness.

## 3. Results and Discussion

This section provides a detailed description of the results obtained from the static and dynamic characterization tests carried out on the IP_6_, IP_9_, and IP_12_ laminate configurations. The findings are thoroughly analyzed, allowing for an evaluation and discussion of the performance and potentialities of the hybrid composites under investigation in this work.

### 3.1. Tensile Test

**Figure 1:**
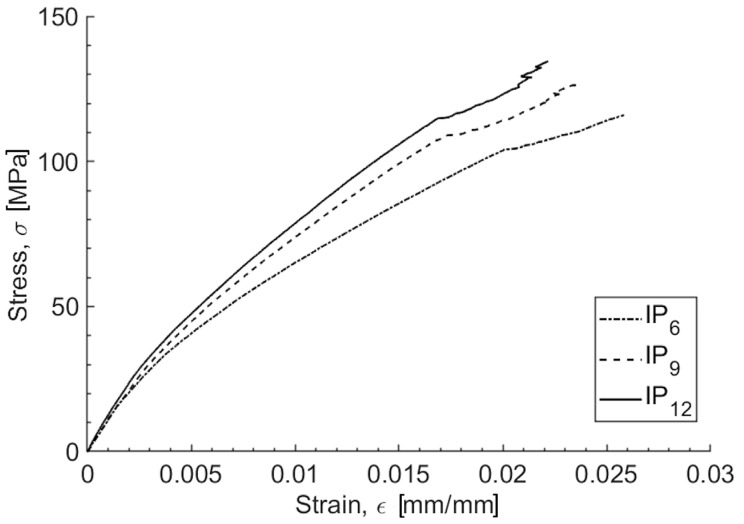
Typical stress–strain trends for the IP_6_, IP_9_, and IP_12_ configurations.

The tensile characterization of the specimens with varying numbers of plies reveals differences in their mechanical behavior. Figure 1 illustrates a representative stress vs. strain trend for each studied configuration. From the literature, flax fiber composites exhibit a stress–strain curve under tensile loading that is hardly linear [27,39]. In contrast, basalt composites tend to display a more linear response [27]. Flax/basalt hybrid composites show a compromise, almost linear behavior, with noticeable load drops, likely caused by the onset of internal damage. The deviation from perfect linearity can be attributed to the interaction between the fibers and the matrix (such as the load transfer, fiber slippage within the matrix, and local debonding), which influences the mechanical response during tensile loading. The mean values, standard deviations, and standard errors are detailed in Table 3 for the tensile modulus *E*, the tensile strength σ_m_, and the maximum deformation ε_max_, as a function of the number of plies. Both the tensile modulus and the tensile strength increase with an increasing number of plies, whereas the maximum strain decreases as the number of plies increases. These observations can be explained by considering the structural reinforcement provided by additional plies. The increase in the number of plies leads to the stiffening of the material, which in this case results in a 28% increase in the tensile modulus and a 15% increase in the tensile strength of the 12-ply laminate configuration, when compared to the 6-ply configuration. The additional plies, however, constrain the deformation, resulting in a reduction in the maximum strain, with a 9% decrease observed for the 12ply configuration compared to the 6-ply configuration.

In Table 4, the F-values and *p*-values obtained from the ANOVA analysis are presented. For the tensile modulus, the ANOVA results show a significant difference among the groups, with an F-value of 4.52 and *p*-value of 0.034 (as detailed in Table 4). This indicates that there are statistically significant differences in the elastic modulus among the IP_6_, IP_9_, and IP_12_ configurations. Tukey’s HSD test further elucidates these differences. The results show that IP_12_ exhibits a significantly higher mean tensile modulus (13,883.8 MPa) compared to IP_9_ and IP_6_. Specifically, IP_12_ is grouped separately as ‘A’, indicating it is significantly different from IP_6_, which is grouped as ‘B’. IP_9_ shares a letter with both IP_6_ and IP_12_, indicating that it is not significantly different from IP_12_, but is significantly different from IP_6_, as detailed in Table 5.

For the tensile strength, the ANOVA results indicate an even stronger significant difference among the groups, with an F-value of 183.69 and a *p*-value of zero (as detailed in Table 4). Tukey’s HSD test confirms that IP_12_ has the highest mean tensile strength (133.38), which is significantly greater than both IP_9_ (125.9 MPa) and IP_6_ (115.3 MPa), grouped as ‘B’ and ‘C’, respectively. IP_9_ and IP_6_ are also significantly different from each other, with IP_6_ having the lowest mean tensile strength. Thus, IP_12_ stands out with a significantly higher tensile strength, while IP_9_ and IP_6_ show progressively lower values, as detailed in Table 5. These results underscore that both the tensile modulus and tensile strength vary significantly across the different groups, with IP_12_ consistently showing superior tensile mechanical properties in comparison to the other tested configurations.

Similar results were obtained by Fragassa et al. [27]. Specifically, they achieved a tensile strength of 86.5 MPa and a tensile modulus of 8151 MPa for a hybrid laminate including flax and basalt fibers in a vinyl ester matrix, with a fiber volume content of 25.5%.

A comparison with the literature reveals that the material under study exhibits superior tensile properties compared to other biocomposites, as revealed below. Prasad et al. [40] investigated the tensile properties of flax fiber-reinforced composites and reported a tensile strength of 65 MPa for a fiber content of 30%, using an optimized laminate production process. Odusote et al. [41] examined the mechanical properties of pineapple leaf fiber-reinforced polymer composites and reported a tensile strength of 80 MPa and a tensile modulus of 8150 MPa for a fiber content of 50%. Yamini et al. [42] investigated the tensile properties of three types of polymer matrix composites reinforced with natural fibers. They claimed that a pure kenaf reinforcement resulted in a tensile strength of 43 MPa, hemp reinforcement yielded 63 MPa, and mixed kenaf + hemp reinforcement achieved 40.5 MPa. These comparisons highlight the enhanced tensile performance of the studied material.

### 3.2. Flexural Test

**Figure 2:**
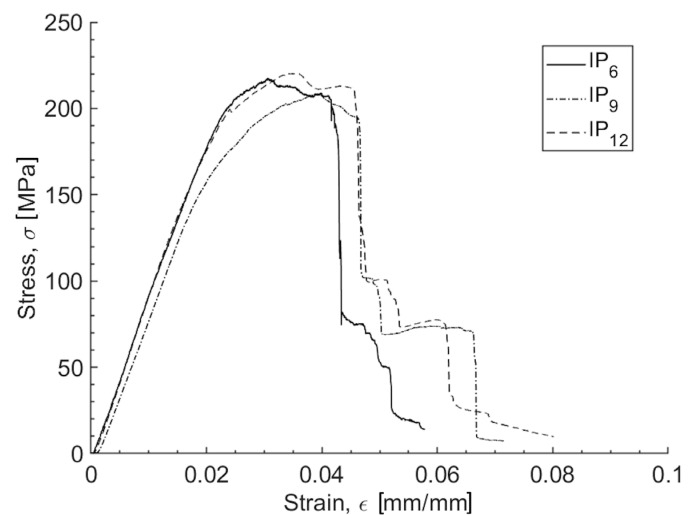
Typical flexural stress–strain trends for the IP_6_, IP_9_, and IP_12_ configurations.

The flexural characterization of the specimens with varying numbers of flax/basalt hybrid plies revealed differences in their mechanical behavior. Figure 2 illustrates the typical bending stress vs. strain trend for each studied configuration. The mean values, standard deviations, and standard errors are detailed in Table 6 for the bending modulus *E*_f_, the flexural strength σ_f_, and the maximum deformation ε_f_,_max_, as a function of the number of plies. Both the flexural modulus and the flexural strength exhibit a V-shaped trend. This means that there is a decrease in both the flexural properties when transitioning from a 6-ply to a 9-ply laminate, followed by an increase as the number of plies increases from 9 to 12. Nevertheless, the slight increase in the bending properties that is observed when the number of plies increases from 9 to 12 still produces a lower flexural modulus and flexural strength than that obtained for the IP_6_ configuration. Therefore, contrary to the observations from the tensile-related characterizations, it can be stated that increasing the number of plies in the laminate does not yield a consistent benefit in terms of the bending modulus and flexural strength.

The ANOVA results for the flexural modulus show an F-value of 2.80 and a *p*-value of 0.10 (as shown in Table 7), which is above the conventional significance level of 0.05. This indicates that there is no statistically significant difference in the flexural modulus among the groups (IP_6_, IP_9_, and IP_12_). This lack of significance is corroborated by Tukey’s HSD test results, which assign all groups with the same letter, ‘A’, as detailed in Table 8. This implies that no group differs significantly from the others in terms of the mean flexural modulus.

For the flexural strength, the ANOVA results yield an F-value of 3.72 with a *p*-value of 0.055 (as shown in Table 7), which is marginally above the conventional significance threshold of 0.05. Although this suggests a near-significant trend, the result is not statistically significant. Tukey’s HSD test, however, shows that all the groups (IP_6_, IP_9_, and IP_12_) are grouped under the same letter, ‘A’, as detailed in Table 8. This indicates that, despite the near-significant *p*-value, there are no statistically significant differences in the mean flexural strength among the groups. In summary, both the flexural modulus and flexural strength analyses suggest that there are no significant differences between the groups for these properties. Tukey’s HSD test reinforces these findings by grouping all the means under the same letter, indicating that no significant differences exist among the tested groups.

Nevertheless, in numerical terms, this outcome highlights the potential flexural performance of the laminate under investigation. Raja et al. [43] created five distinct composite laminates using a hand layup technique, including flax and basalt fibers reinforced with zinc oxide particles mixed into an epoxy polymer matrix to enhance thermal resistance. They reported that the sample with higher basalt fiber content exhibited superior flexural strength, measuring 81 MPa, which is less than half the flexural strength of the laminate studied in this work.

### 3.3. Charpy Test

**Figure 3:**
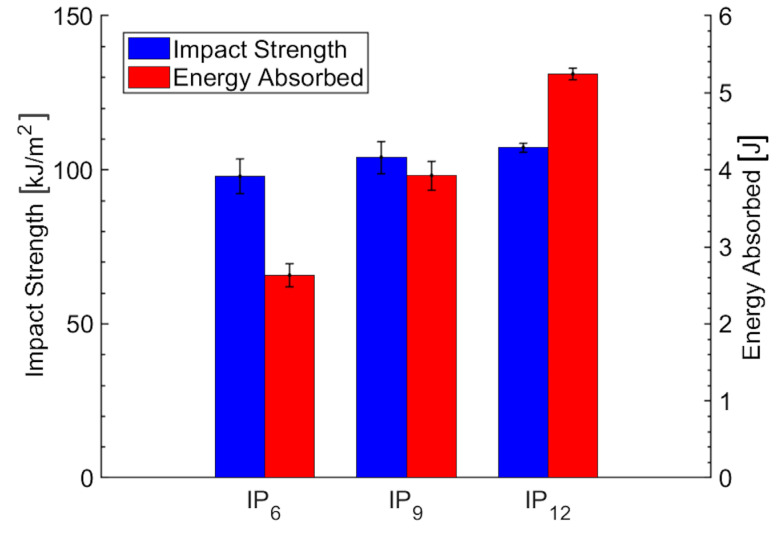
Impact strength and energy absorbed as a function of the number of plies.

During the Charpy impact testing of laminated composites, an increase in the number of plies, from 6 to 12, was observed to enhance the absorbed energy, as shown in Figure 3. The mean values, standard deviations, and standard errors are detailed in Table 9. Regarding the impact strength, although a slight graphical increase is observed with the increasing number of plies, the numerical data (as shown in Table 9) does not consistently support this trend.

Based on the ANOVA and Tukey’s HSD test results for the impact strength and the absorbed energy, the following observations can be made. The ANOVA results for the impact strength show an F-value of 1.09 and a *p*-value of 0.367 (as reported in Table 10), which is well above the significance level of 0.05. This indicates that there is no statistically significant difference in the impact strength among the groups (IP_6_, IP_9_, and IP_12_). Tukey’s HSD test results support this conclusion, as all the groups (IP_6_, IP_9_, and IP_12_) are assigned the same letter, ‘A’, as detailed in Table 11. This implies that, according to Tukey’s test, there are no significant differences in the mean impact strength across the groups.

The ANOVA results for the absorbed energy reveal an F-value of 80.89 and a *p*-value of zero (as reported in Table 10), indicating highly significant differences among the groups. Tukey’s HSD test results further clarify these differences, showing that IP_12_ has a mean impact energy of 5.24, which is significantly higher than IP_9_ and IP_6_. Specifically, IP_12_ is grouped as ‘A’, IP_9_ as ‘B’, and IP_6_ as ‘C’, as detailed in Table 11. This grouping indicates that IP_12_ has significantly greater impact energy compared to IP_9_ and IP_6_, and IP_9_ has significantly greater impact energy than IP_6_.

**Figure 4:**
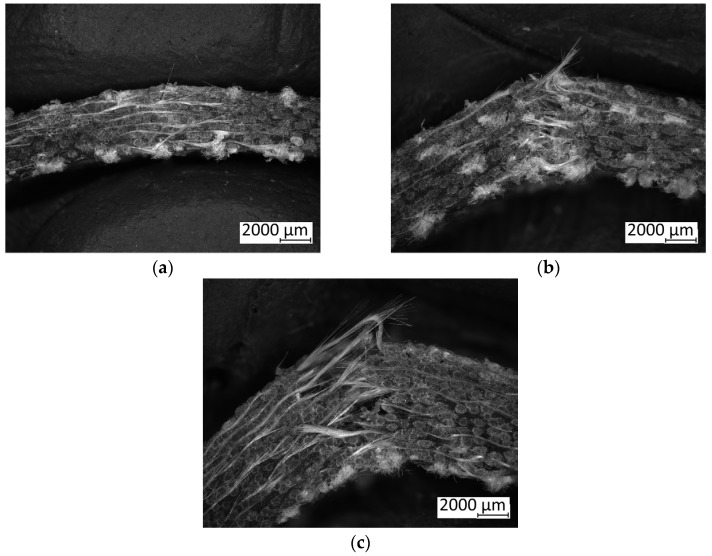
Microscopic detail of Charpy test specimens, labelled as follows: (**a**) IP_6_; (**b**) IP_9_; and (**c**) IP_12_.

However, this improvement in the mechanical properties is accompanied by a significant increase in delamination, as evidenced by the microscopic examinations, as shown in Figure 4. The increase in the ply count results in a thicker laminate with a greater overall energy absorption capacity, due to the increased material volume available to dissipate the impact forces. Despite this, the tendency for delamination also rises with the increasing number of plies. This is attributable to the increasing complexity of the internal structure and the presence of a larger number of interfaces between the individual plies, which can weaken adhesive bonds and create more potential sites for failure. On the other hand, the increase in absorbed energy can also be attributed to a higher likelihood of increased porosity at the interfaces between the layers with increasing number of plies, which creates preferred sites for energy absorption. Although thicker laminates with more plies can absorb more total energy before fracture, the onset of delamination indicates a critical trade-off. The energy absorbed is partly used in separating the layers rather than deforming the material, which compromises the laminate’s structural integrity and impact resistance (see Figure 4b,c). Thus, while adding plies improved the energy absorption and impact strength of the laminates under study, it also necessitates careful consideration of the adhesive bonding quality and manufacturing processes to mitigate delamination issues.

A comparative analysis was conducted with a case study from the literature. Yeter et al. [44] investigated the impact properties of basalt/epoxy and carbon/epoxy laminates using Charpy flatwise impact testing, obtaining average absorbed energies of 1.95 J and 1.28 J, respectively. The absorbed energy of 1.95 J represents the average value obtained from three specimens consisting of 6, 9, and 12 plies for basalt/epoxy laminates. Similarly, the absorbed energy of 1.28 J is the average value obtained from three specimens with 6, 9, and 12 plies for carbon/epoxy laminates. In contrast, the laminate investigated in this study, with the minimum ply thickness (IP_6_), absorbs 2.63 J of energy. This represents a 35% increase, compared to the basalt/epoxy laminate and a 105% increase compared to the carbon/epoxy laminate reported by Yeter et al. [44].

### 3.4. Drop-Weight Test

**Figure 5:**
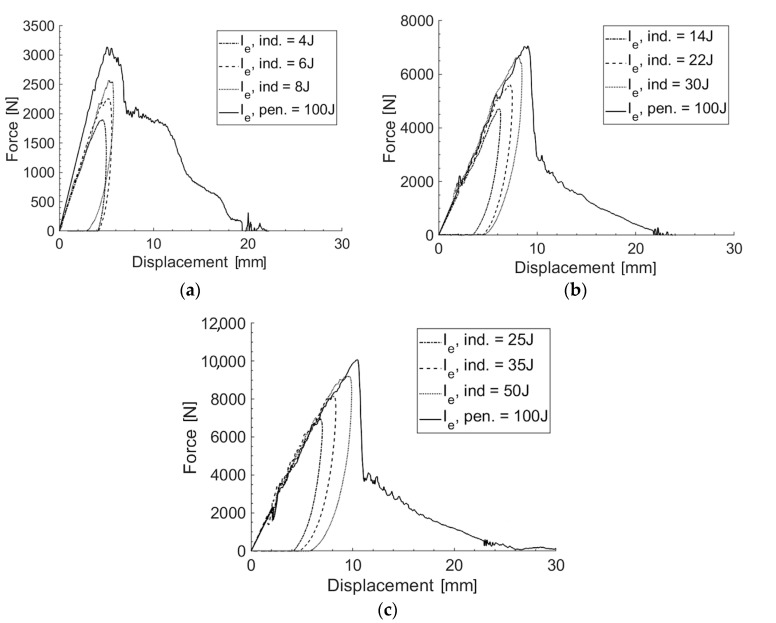
Force vs. displacement penetration and indentation curves for different levels of impact energy: (**a**) IP_6_; (**b**) IP_9_; and (**c**) IP_12_.

**Figure 6:**
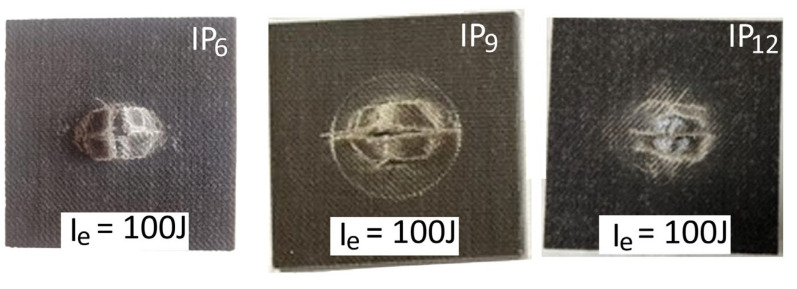
Pictures of IP_6_, IP_9_, and IP_12_ specimens impacted by the same level of energy, I_e_, at penetration.

**Figure 7:**
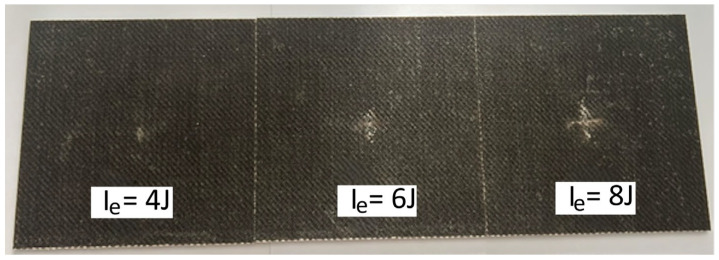
Pictures of the indentation damage that occurred on the IP_6_ specimens, impacted with three different levels of impact energy I_e_.

In the low-velocity drop-weight impact tests, laminates with 6, 9, and 12 plies were preliminarily characterized for penetration. Figure 5 illustrates the force versus displacement behavior for each laminate configuration at various impact energy levels, encompassing both indentation and penetration tests. Figure 6 further shows the detail of the damage caused by the penetration impact test. The results demonstrated a clear trend in the energy absorption capabilities of the laminates. As the number of plies increased, the absorbed energy at penetration significantly increased (see Table 12). This indicates that thicker laminates, with more plies, possess higher resistance to penetration and can absorb a higher amount of impact energy before failure. However, it is worth noting from the penetration force–displacement curves in Figure 7 that the IP_6_ laminate configuration, consisting of six plies, after an initial drop in the impact force indicative of an initial damage phase, continues to sustain the load, creating an almost plateau-like behavior at a force level of approximately 1000 N below the peak force, prior to complete damage, i.e., before the force drops to zero. This plateau behavior is less evident for the IP_9_ and IP_12_ configurations. This means that in the case of IP_9_ and IP_12_, the failure can be considered catastrophic, while the IP_6_ configuration would result in a stepwise failure and, thus, provide warning indications prior to complete failure.

This is consistent with the findings reported by Ricciardi et al. [45]. They studied the effect of hybridization on the impact properties of flax/basalt epoxy composites. The behavior observed in terms of the load–displacement curve at penetration aligns with the results obtained in the present work. Furthermore, these observations align with those reported by Papa et al. [46], indicating that the use of basalt fibers can increase impact energy absorption compared to carbon fiber alternatives. 

By observing Figure 6, which illustrates the details of the laminates after the penetration impact test, the specimen consisting of six plies, based on visual inspection, shows a less extensive overall damage area compared to the IP_9_ and IP_12_ configurations. Specifically, the laminates with 9 and 12 plies exhibit a circular crown around the main damage area, characterized by additional cracks and delamination.

When comparing the performance under indentation at various impact energy levels, a similar trend was observed. The 12-ply laminate exhibited the highest maximum load and absorbed energy across all the tested impact energy levels, followed by the 9-ply and 6-ply laminates (see Table 13 and Figure 5). This suggests that the additional plies contribute to an enhanced load-bearing capacity and energy dissipation mechanism, likely due to the increased material volume and improved distribution of stress and strain within the laminate structure.

A two-way ANOVA analysis was conducted to evaluate the effect of the laminate configuration and impact energy (coded as 0 for low, 1 for medium, and 2 for high) on the absorbed energy at penetration response. The results indicate that the laminate configuration (IP_6_, IP_9_, and IP_12_) has no impact, as the *p*-value is well below the standard significance level of 0.05. In contrast, the impact energy shows a marginal effect on the absorbed energy, with the *p*-value slightly above the 0.05 threshold (as detailed in Table 14). Overall, these findings suggest that while the laminate configuration significantly influences energy absorption, the impact energy levels might also have an effect that approaches statistical significance. The difference in the energy absorption and maximum load between the laminates can be attributed to the inherent structural advantages provided by the additional plies. More plies result in a thicker laminate, which not only increases the stiffness and strength of the laminate, but also provides more material to absorb and dissipate the impact energy through mechanisms such as fiber breakage, matrix cracking, and delamination. Consequently, the 12-ply laminates offer superior performance in both the penetration and indentation tests.

**Figure 8:**
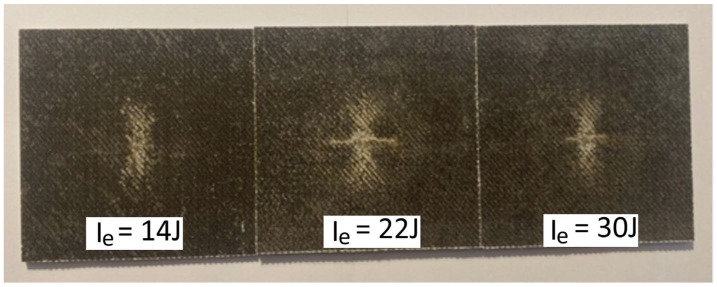
Pictures of the indentation damage that occurred on the IP_9_ specimens, impacted with three different levels of impact energy I_e_.

**Figure 9:**
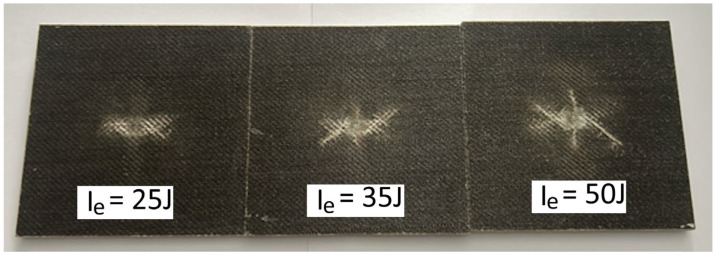
Pictures of the indentation damage that occurred on the IP_12_ specimens, impacted with three different levels of impact energy I_e_.

**Figure 10:**
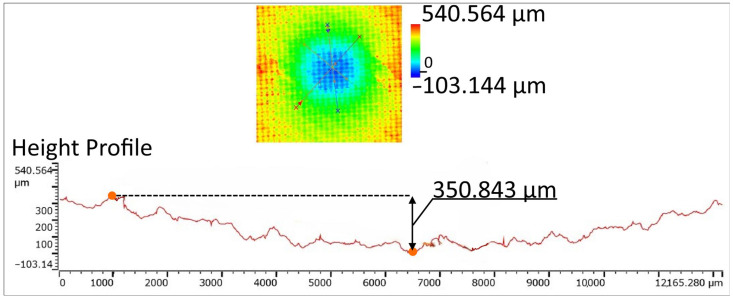
Example of indentation damage depth measurement (IP_12_, I_e_ = 25 J).

For the indentation impact tests, Figure 7, Figure 8 and Figure 9 show the surface damage, corresponding to the IP_6_, IP_9_, and IP_12_ configurations, respectively. These figures illustrate the damage produced by the indentation impact test for each level of impact energy tested. To quantify the damage, as detailed in the Materials and Methods Section, a confocal microscope was used. For illustrative purposes, the output of the confocal microscopy for the IP_12_ specimen, impacted with 25 J of energy, is presented in Figure 10.

**Figure 11:**
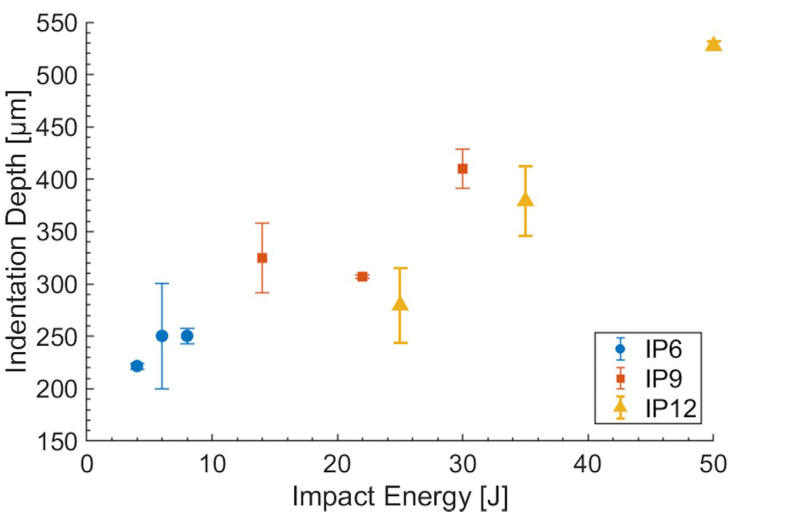
Variation in the indentation damage depth as a function of impact energy for each lamination configuration IP_6_, IP_9_, and IP_12_.

The results from the indentation impact tests reveal distinct patterns in the relationship between the impact energy, absorbed energy, and indentation depth, across the three laminate configurations, IP_6_, IP_9_, and IP_12_, as shown in Figure 11. For the IP_6_ configuration, the indentation depth increases progressively with increasing impact energy. Specifically, the indentation depth shows an increase of 13% from 4 J to 6 J, which then plateaus at 8 J, despite the increase in impact energy. 

In contrast, the IP_9_ configuration exhibits a more pronounced increase in the indentation depth with increasing impact energy. Here, the indentation depth increases by approximately 14% from 14 J to 22 J and by a further 34% from 22 J to 30 J. This suggests that the additional plies in the IP_9_ configuration provide increased resistance to indentation, but this resistance diminishes as the impact energy continues to rise. However, this is contrary to what is commonly found in the literature on traditional fiber laminates [47]. As the thickness increases, the shear effect becomes more significant than the bending effect when the laminate is impacted by a concentrated load [48,49]. This shear effect occurs between the layers, causing delamination [50]. When the thickness is below a certain a threshold limit, most of the absorbed energy is used to bend the component, resulting in less delamination, due to the reduced energy transferred to this type of damage. This indicates a different damage mechanism and different relationships among the various types of damage. Nevertheless, the existence of a threshold thickness governing these damage mechanisms is interesting and it represents a crucial area for further investigation.

The IP_12_ configuration shows the greatest indentation depth across the tested impact energies. The indentation depth increased significantly, with a 36% rise from 25 J to 35 J and a further 39% increase from 35 J to 50 J. This pattern indicates that the IP_12_ configuration, despite having the highest number of plies, absorbs more energy and shows a greater indentation depth as the impact energy escalates, reflecting a greater cumulative response to higher impact forces.

As stated in the Introduction Section, new environmental regulations and evolving governmental attitudes are a powerful, key driver, stimulating the research into more environmentally friendly products and processes. Thus, there is a large amount of interest and considerable research activities are dedicated to finding solutions to minimize the environmental impact of the production process and use of composite materials, therefore leading to an improvement in the sustainability of such materials. As a form of reinforcement, natural fibers (such as flax, hemp, kenaf, wood, bamboo, etc.) are largely investigated as an alternative, involving total or partial substitution, to synthetic fibers (mainly glass fibers, since carbon and aramid offer superior mechanical performance) [51]. Based on the results obtained in this work, basalt is confirmed to be a valid alternative to conventional glass fibers in terms of its good mechanical performance, but also because of its good corrosion resistance. Moreover, basalt fibers also represent a sustainable and cost-effective alternative to glass fibers. 

Further, flax fiber is a natural fiber, with low mechanical properties, but which has a lower density in comparison to synthetic fibers. According to the results obtained, this work confirms that hybridization can be a suitable solution, since the properties of a hybrid composite composed of two fibers are influenced not only by the individual fiber characteristics, but also by the layering pattern of the two fiber types, their intermingling capacity, the fiber-to-matrix interface, and also the failure strain of single fibers. Thus, the design of hybrid composites can be crucial, as well as being very convenient for specific applications [52].

Sustainability is currently imperative in terms of the need to address global challenges, so it needs to be considered in terms of the life cycle aspects of materials, as well as during the manufacturability and functionality of the parts of products. Further research is needed to find an even better and more convenient compromise between the mechanical properties of materials required for specific applications and the reduction needed in terms of the overall environmental impact. This work demonstrates that natural fiber composites could represent a valid solution to pursue these objectives. In particular, it demonstrates the possibilities and opportunities offered not only in terms of the adoption of natural fibers as a substitute for synthetic ones, but also in regard to the possibilities offered in finding new strategies to reduce the impact on the environment, without sacrificing a lot of the mechanical properties. This work proposes a reduction in the thickness of a specific hybrid composite, and a lot of research should be conducted to find new materials, new hybrid composites, and new strategies to pursue this multi-objective purpose.

## 4. Conclusions

This study investigated the possibility of reducing the thickness of hybrid composites reinforced with flax and basalt, without sacrificing the mechanical properties, by fixing the stacking sequence and varying the number of plies. Moreover, a comparison with the findings in the literature demonstrated the potential of the hybrid composites under investigation, which exhibit superior mechanical properties compared to other hybrid composites that have been previously reported. The main conclusions that can be drawn from this research are the following:

- The tensile tests showed a slight increase in the tensile modulus and tensile strength with an increased number of plies, from 6 to 12 plies, thereby increasing the thickness of the laminate from 2.50 mm to 4.70 mm;
- The flexural behavior of the hybrid composites showed that increasing the number of plies did not yield a consistent benefit in terms of the flexural modulus and flexural strength;
- The Charpy tests revealed a slight enhancement in the impact strength with an increase in the number of plies. Nevertheless, delamination phenomena were observed in all the specimens with the maximum thickness (12 plies);
- The drop-weight tests demonstrated a clear trend in the energy absorption of the laminates. In particular, it was observed that energy absorbed at both penetration and indentation significantly increased for thicker laminates, indicating higher resistance to penetration due to its ability to absorb greater impact energy before damage occurs;
- According to the results obtained from the drop-weight tests, the indentation depth increased significantly with the impact energy, showing a 36% increase from 25 J to 35 J, and a further 39% increase was observed when going from 35 J to 50 J;
- The results confirm the potential of basalt fibers as a valid substitute for glass fibers for composite reinforcement. Basalt fibers offer several benefits in terms of their mechanical properties, while also allowing for weight and cost reductions, thus enhancing the sustainability of the manufacturing process and the life cycle of the fabricated laminates;
- Finally, the overall results of this experimental study demonstrated that the hybrid composites under investigation, made with a green epoxy resin reinforced with flax and basalt, can have a reduced laminate thickness, by up to 47%, without sacrificing the tensile, flexural, and resilience properties;
- Even though the drop-weight tests showed that a greater thickness leads to better impact responses, thinner hybrid composite laminates can be optimal for certain structural applications, but are probably not ideal for impact-loading applications.

Further steps in investigating this innovative biocomposite may involve the implementation of a numerical model to forecast the impact behavior as a result of specific stacking sequences. Additionally, further research is needed to deeply understand and address the delamination mechanisms that occur in thicker laminates. Fatigue tests may also provide a deeper understanding of the mechanical behavior of this hybrid composite material under specific loading conditions. Moreover, data on the defectiveness of the initial structure and crack propagation would be useful to investigate additional possibilities offered by the hybrid composite investigated in this study, as well as its limitations due to the requirement for other physical and chemical treatments to face other eventual challenges. Finally, new combinations of resins and hardeners, as well as hybrid natural fiber plies, should be developed from a sustainability perspective.

## Figures and Tables

**Table 1:** Hybrid laminate design.

Stacking Sequence	Number of Plies	Total Fiber Weight [g]	Total Laminate Weight [g]	Fiber Mass Fraction [%]	Length [mm]	Width [mm]	Thickness [mm]
[IP_6_]	6	351	791.70	44.3	670	300	2.50 ± 0.20
[IP_9_]	9	543	1,172.13	46.3	670	300	3.70 ± 0.20
[IP_12_]	12	721	1,525.77	47.3	670	300	4.70 ± 0.20

**Table 2:** Details of the penetration and indentation energies used for impacting IP_6_, IP_9_, and IP_12_ laminates.

Impact Energy at Penetration [J]		
IP_6_	IP_9_	IP_12_
100	100	100
**Impact Energy at Indentation [J]**		
**IP_6_**	**IP_9_**	**IP_12_**
4	14	25
6	22	35
8	30	50

**Table 3:** Summary of tensile test results.

	E [MPa]	σ_m_ [MPa]	ε_max_ [mm/mm]
	IP_6_		
Mean value	10,823.9	115.3	0.0232
St. Deviation	376.2	1.1	0.0030
St. Error	168.2	0.5	0.0013
	**IP_9_**		
Mean value	12,040.3	125.9	0.0225
St. Deviation	1,927.7	1.6	0.0014
St. Error	862.1	0.7	0.0006
	**IP_12_**		
Mean value	13,883.8	133.3	0.0211
St. Deviation	2,002.7	1.7	0.0014
St. Error	895.6	0.7	0.0006

**Table 4:** Analysis of variance in terms of tensile test outputs.

	Tensile Modulus, *E*	Tensile Strength, σ_m_
F-value	4.52	183.69
*p*-Value	0.034	0

**Table 5:** Mean values and statistical groupings for tensile modulus and tensile strength.

	Tensile Modulus, *E*		Tensile Strength, σ_m_	
	Mean [MPa]	Group	Mean [MPa]	Group
IP6	10,823.9	B	115.3	C
IP9	12,040.3	A and B	125.9	B
IP12	13,883.8	A	133.3	A

**Table 6:** Summary of bending test results.

	*E*_f_ [MPa]	*σ*_f_ [MPa]	ε_f_,_max_ [mm/mm]
	IP_6_		
Mean value	8,994.9	216.3	0.0618
St. Deviation	491.9	5.9	0.0097
St. Error	219.9	2.6	0.0043
	**IP_9_**		
Mean value	7,675.7	194.7	0.0778
St. Deviation	1,146.0	18.9	0.0091
St. Error	512.5	8.4	0.0040
	**IP_12_**		
Mean value	7,660.3	215.6	0.0714
St. Deviation	2,017.3	9.3	0.0067
St. Error	902.1	4.2	0.0030

**Table 7:** Analysis of variance in terms of bending test outputs.

	Flexural Modulus, *E*_f_	Flexural Strength, σ_f_
F-value	2.80	3.72
*p*-Value	0.10	0.055

**Table 8:** Mean values and statistical groupings for flexural modulus and flexural strength.

	Flexural Modulus, *E*_f_		Flexural Strength, σ_f_	
	Mean [MPa]	Group	Mean [MPa]	Group
IP6	8,994.9	A	216.3	A
IP9	7,675.7	A	194.7	A
IP12	7,660.3	A	215.6	A

**Table 9:** Summary of Charpy test results.

	Impact Strength [kJ/m^2^]	Energy Absorbed [J]	
IP_6_			
Mean value	97.9	2.63	
St. Deviation	12.42	0.33	
St. Error	5.55	0.15	
**IP_9_**			
Mean value	103.9	3.92	
St. Deviation	11.68	0.41	
St. Error	5.22	0.18	
**IP_12_**			
Mean value	107.1	5.24	
St. Deviation	3.40	0.17	
St. Error	1.52	0.08	

**Table 10:** Analysis of variance in terms of Charpy test outputs.

	Impact Strength	Energy Absorbed
F-value	1.09	80.89
*p*-Value	0.367	0

**Table 11:** Mean values and statistical groupings for the impact strength and the energy absorbed.

	Impact Strength		Energy Absorbed	
	Mean [kJ/m^2^]	Group	Mean [J]	Group
IP6	97.9	A	2.63	C
IP9	103.9	A	3.92	B
IP12	107.1	A	5.24	A

**Table 12:** Impact energy and corresponding absorbed energy at penetration for IP_6_, IP_9_, and IP_12_ specimens.

	Impact Energy, I_e_ [J]	Absorbed Energy at Penetration, E_p_ [J]
IP_6_	100	27.20
IP_9_	100	52.40
IP_12_	100	84.20

**Table 13:** Details of the maximum impact force and the absorbed impact energy during the indentation of all the configurations under investigation at different impact energy levels.

	Impact Energy, I_e_ [J]	Maximum Load [N]	Absorbed Energy at Indentation, E_i_ [J]
IP_6_	4	1,900.7	3.64
	6	2,253.4	5.52
	8	2,566.9	7.95
IP_9_	14	4,702.7	13.71
	22	5,623.6	21.70
	30	6,642.5	29.87
IP_12_	25	6,956.1	24.12
	35	8,151.3	36.82
	50	9,209.4	49.47

**Table 14:** Analysis of variance in terms of drop-weight test outputs.

		Absorbed Energy at Indentation
Laminate configuration		
	F-value	26.08
	*p*-Value	0.005
Impact Energy		
	F-value	6.29
	*p*-Value	0.058

## Data Availability

The data are contained within the article.
